# Assessment of Mortality Disparities by Wealth Relative to Other Measures of Socioeconomic Status Among US Adults

**DOI:** 10.1001/jamanetworkopen.2022.6547

**Published:** 2022-04-08

**Authors:** Dana A. Glei, Chioun Lee, Maxine Weinstein

**Affiliations:** 1 Center for Population and Health, Georgetown University, Washington, DC; 2 Department of Sociology, University of California, Riverside

## Abstract

**Importance:**

The association between wealth and mortality is likely to be nonlinear and may result from selection and reverse causality.

**Objective:**

To compare the magnitude of mortality disparities by wealth relative to other measures of socioeconomic status (SES).

**Design, Setting, and Participants:**

This population-based cohort study began in 1995 to 1996, with approximately 18 years of mortality follow-up. These analyses were completed in November 2021. Data were derived from a population-based sample that targeted noninstitutionalized, English-speaking adults aged 25 to 74 years in the contiguous US. The response rate for the telephone interview ranged from 60% (twin subsample) to 70% (main sample). A self-administered questionnaire was completed by 89% of those interviewed by telephone.

**Exposures:**

Net assets of the respondent and spouse or partner in 1995 to 1996.

**Main Outcomes and Measures:**

All-cause mortality.

**Results:**

Among 6320 respondents (mean [SD] age at baseline, 46.9 [12.9] years; 3318 women [52.5%]), 1000 (15.8%) died by May 31, 2013. Adjusted for age, sex, and race, the mortality disparity by wealth was larger than the disparities by education, occupation, income, or childhood SES, especially at the oldest ages. After age 65 years, the hazard ratio [HR] was 2.69 (95% CI, 2.00-3.62) for those with no assets relative to those with at least $300 000 of wealth (in 1995 dollars), which translated into a 31 percentage point differential in estimated probability of surviving from age 65 years to 85 years (40% vs 71%). Additional wealth greater than $500 000 was not associated with lower mortality. In fully adjusted models, there was still a sizeable wealth disparity in mortality after age 65 years (HR, 1.89; 95% CI, 1.33-2.67). After adjustment for confounders, the estimated probability of surviving from age 65 to 85 years was 19 percentage points higher for persons with at least $300 000 in wealth (70%) than for those with no assets (51%), but there was a much larger 37 percentage point differential between never smokers (70%) and current smokers (33%).

**Conclusions and Relevance:**

In this cohort study, the fully adjusted disparity in mortality associated with wealth beyond age 65 years remained sizeable but was much smaller than the smoking differential.

## Key Points

**Question:**

How does the disparity in mortality by wealth compare with disparities by other socioeconomic status (SES) measures and by smoking history?

**Findings:**

In this US cohort study involving 6320 participants, disparities in mortality associated with wealth were statistically significant and larger than the disparities associated with education, occupation, income, or childhood SES; at high levels, additional wealth was not associated with lower mortality. However, the wealth disparity was much smaller than the smoking differential, which was evident across all ages 25 years and older.

**Meaning:**

These findings suggest that mortality disparities by wealth are larger than those associated with other indicators of SES but that the smoking differential is far greater.

## Teaser

This cohort study compares the magnitude of mortality disparities associated with wealth with disparities associated with other measures of socioeconomic status.

## Introduction

The positive association between socioeconomic status (SES) and longevity is well established,^1,2,3^ and although much of the literature relies on education or income as a proxy measure for SES, some studies have focused on wealth.^4,5,6,7,8^ Because both income and wealth inequality have increased substantially since the late 1980s,^9,10^ examining wealth as a factor associated with differential longevity has also become more salient.

Here we consider 3 crucial factors that affect the association between wealth and longevity. First, the inverse association between wealth and mortality is nonlinear: the mortality disparity associated with a fixed difference in wealth is much greater among the poor than among the rich.^11^ Although a recent study^5^ reported a surprisingly small association between wealth and longevity, we suspect it was because they assumed the association was linear, resulting in underestimating the mortality disparity at low levels of wealth, but overestimating the survival differential at high levels of wealth.

Second, the relative importance of different measures of SES varies by life stage. In early adulthood, educational attainment is a critical determinant of occupation, which, in turn, is associated with income and wealth accumulation. During midlife, income and wealth influence one’s ability to buy a home, which further affects wealth accumulation. After retirement, education and occupation become less salient for health, whereas income becomes more dependent on wealth. Thus, wealth is likely to be the most important determinant of the SES differential in mortality at the oldest ages. Some studies found that wealth estimates mortality better than other SES measures,^8,12,13^ but only 1 of these studies included anyone younger than 50 years.^13^ That study found that, among those aged 45 to 64 years, income and wealth were associated with greater differences in mortality compared with education or occupation but wealth was, by far, the most important factor after age 65 years.^13^ Another study suggested that disparities in mortality associated with wealth were larger at older ages (70-80 years) than in late midlife (55-64 years),^11^ which contrasts sharply with research^13,14,15^ suggesting that disparities in mortality associated with SES tend to diminish with age and with some other studies^7,8,13^ showing a similar pattern for wealth. Few studies have investigated whether the difference in mortality associated with wealth varies across the life course.

Finally, whether wealth has a causal effect on mortality remains controversial.^11^ The overall wealth-related disparity in mortality does not imply causation; it simply reflects the degree of inequality. The disparity may be a result of selection and/or reverse causality. Medical researchers often assume that wealth increases longevity, whereas many economists suspect that wealth depends on health.^16,17^ For example, serious illness may inhibit wealth accumulation and medical bills can decimate accumulated wealth. Thus, it is critical to account for a wide array of confounders, including health status.

Using a cohort aged 20 to 75 years with 18 years of mortality follow-up, we examine 3 questions. Does the survival differential associated with a given increment in wealth decrease at higher levels of wealth? Does the importance of wealth compared with other measures of SES increase with age? Do wealth disparities persist after adjusting for potential confounders? To better contextualize the relative importance of wealth for mortality, we compare it with smoking history, which demonstrated the strongest association with mortality among 57 economic, behavioral, social, and psychological factors.^18^

## Methods

### Data

The data come from the Midlife in the United States (MIDUS) study, which targeted noninstitutionalized, English-speaking adults aged 25 to 74 years in the contiguous US.^19^ (Although the sampling frame targeted adults aged 25-74 years, the final sample included 15 respondents aged 20-24 years and 4 respondents aged 75 years at the baseline telephone interview.) The protocol for MIDUS was approved by the institutional review board at the University of Wisconsin-Madison. Written, informed consent was obtained from all study participants. We followed the Strengthening the Reporting of Observational Studies in Epidemiology (STROBE) reporting guidelines for cohort studies.

At baseline (fielded January 1995 to September 1996), national random digit dialing with oversampling of older people and men was used to select the main sample (3487 individuals) and a sample of twin pairs (1914 individuals). The study also included a random subsample of siblings of individuals in the main sample (950 individuals) and oversamples from 5 metropolitan areas in the US (757 individuals). The response rate for the telephone interview ranged from 60% for the twin subsample to 70% for the main sample. Among the 7108 individuals who completed the telephone interview, 6325 (89%) also completed mail-in self-administered questionnaires. We analyzed deaths through May 31, 2013, beyond which mortality follow-up may be incomplete (see eAppendix 1 in the Supplement for details).

### Measures

Vital status was ascertained through searches of the National Death Index, survey fieldwork, and longitudinal sample maintenance (see eAppendix 1 in the Supplement for details).^20^ All covariates were measured at baseline (1995-1996). Wealth was based on the current net assets of the respondent and their spouse or partner. Other SES-related measures included educational attainment, occupational socioeconomic index (SEI),^21^ household income, an index of overall adult SES, and an index of childhood SES (see eAppendix 2 and eTable 1 in the Supplement).

We included potential confounders that could affect wealth accumulation and are known to be associated with mortality. Demographic confounders comprised sex, age, and race. Respondents were asked, “What race do you consider yourself to be?” We retained the first 2 response categories (ie, Black and/or African American and White), but combined the remaining categories (ie, Asian or Pacific Islander; multiracial; Native American, Aleutian Islander, or Eskimo; and other) into a group labeled other race. Race was assessed in this study because racial disparities in wealth and mortality are well established. Socioeconomic variables included marital status, health insurance coverage, and employment status. Health-related confounders encompassed parental health status, the respondent’s preexisting health conditions, and spouse’s or partner’s health status. Finally, we adjusted for substance use. See eTable 2 in the Supplement for more details about the confounders.

### Statistical Analysis

We used standard practices of multiple imputation to handle missing data^22,23^ (see eAppendix 3 in the Supplement for more details). Statistical tests were 2-sided at the *P* < .05 level. All analyses were conducted using Stata statistical software version 16.1 (StataCorp). These analyses were completed in November 2021.

We modeled age-specific mortality using a Cox model with age as the time metric and used a robust variance estimator to correct for family-level clustering. Previous studies^5,7,8,11,13^ used duration of study as the time metric with a control for age at baseline, which they treated as linear. Given that age is a time-varying covariate and yields, by far, the highest discriminatory ability for distinguishing between those who survived vs died within 5 years,^24^ we prefer to use age as the clock, which allows the baseline hazard to follow any functional form over age.

To evaluate the overall magnitude of SES disparities, our initial models adjusted only for age, sex, and race. Because Black vs White disparities in mortality diminish with age, we included an interaction between age and Black race. To demonstrate absolute differences in survival, we plotted the estimated probability of survival from age 25 to 75 years by level of wealth with all other covariates fixed at the mean.

There was no evidence that wealth disparities varied by age, but we found nonproportional hazards for many of the other SES measures. Therefore, we estimated the remaining models separately for mortality before and after age 65 years. Within those restricted age intervals, there was no evidence of nonproportional hazards for any of the covariates. On the basis of the 2 models for mortality at ages younger and older than 65 years, we plotted the survival probabilities from age 25 to 65 years and from age 65 to 85 years, respectively.

In the final set of models, we adjusted for all the potential confounders listed in eTable 2 in the Supplement. The model for each SES measure also adjusts for the other SES measures that are temporally prior (ie, education is assumed to precede occupation; education and occupation precede current income and current wealth).

## Results

**Figure 1.**
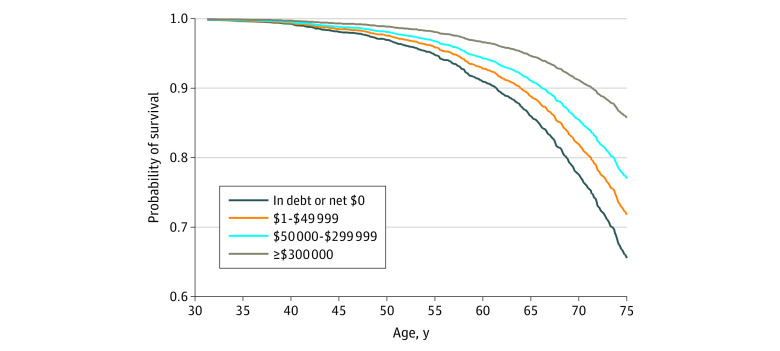
Demographic-Adjusted Probability of Surviving From Age 25 to 75 Years by Level of Wealth There were no deaths before age 30 years. The estimated survival curves are based on a model of age-specific mortality that controls for sex and race; those variables are fixed at the mean for the sample. The Black-White racial differential in mortality was allowed to vary by age (ie, nonproportional hazards).

We excluded 5 respondents for whom the date of death was unknown, leaving an analysis sample of 6320 respondents who completed the self-administered questionnaire at baseline, 1000 (15.8%) of whom died by May 31, 2013. The mean (SD) age of the cohort at baseline was 46.9 (12.9) years, and 36% of the sample was aged 40 years or younger at baseline; 3318 (52.5%) were women; 5709 (90.3%) identified as White, 342 (5.4%) identified as Black, and 269 (4.3%) identified as some other race (eTable 2 in the Supplement). Table 1 shows the overall wealth disparities in mortality, adjusted only for age, sex, and race. Although the hazard ratio (HR) for those with zero net assets appeared to be even higher than for those in debt, the difference was not significant (model 1). Thus, we combined those 2 groups in model 2. Although mortality rates generally declined as assets increased, the association was nonlinear. The first $50 000 in assets reduced the mortality rate by 22% (model 2), whereas the next $50 000 produced a nonsignificant 17% decrease (0.65/0.78 = 0.83; 95% CI, 0.65-1.06), and another $50 000 yielded a nonsignificant 5% reduction (0.61/0.65 = 0.95; 95% CI, 0.71-1.27). In fact, we found no significant differences across groups with $50 000 to $299 999 in wealth. However, those with $300 000 to $499 999 had 41% lower mortality rates than those with only $200 000 to $299 999 (0.34/0.58 = 0.59; 95% CI, 0.40-0.85). Above $500 000, additional wealth yielded no mortality dividend (ie, the differences across the top 3 categories of wealth, comprising all those with at least $300 000, were not significant). Thus, in model 3, we collapsed wealth into 4 categories (ie, debt or no assets, $1-$50 000, $50 000-$299 999, and $300 000 or more). Figure 1 translates the results from model 3 into the absolute estimated probability of surviving from age 25 to 75 years, which was 20 percentage points lower for those with no assets or debt (66%) than for their counterparts with at least $300 000 (86%).

**Table 1.** HRs for Wealth as a Factor Associated With Age-Specific Mortality, Adjusted for Demographic Characteristics Only, Among 6320 Participants in the Midlife in the United States Study, 1995-2013

Variable	HR (95% CI)		
	Model 1	Model 2	Model 3
Female sex	0.64 (0.56-0.74)	0.65 (0.57-0.74)	0.65 (0.57-0.74)
Race			
Black^a^	4.74 (1.56-14.43)	4.76 (1.57-14.49)	4.76 (1.60-14.17)
Age and Black race interaction	0.97 (0.95-0.99)	0.97 (0.95-0.99)	0.97 (0.95-0.99)
White	1 [Reference]	1 [Reference]	1 [Reference]
Other race^b^	0.86 (0.58-1.29)	0.87 (0.58-1.30)	0.87 (0.58-1.29)
Wealth, $^c^			
In debt	1 [Reference]	1 [Reference]	1 [Reference]
Net 0	1.16 (0.83-1.61)	1 [Reference]	1 [Reference]
1-49 999	0.87 (0.64-1.18)	0.78 (0.64-0.95)	0.78 (0.64-0.95)
50 000-99 999	0.72 (0.51-1.00)	0.65 (0.51-0.83)	0.62 (0.51-0.74)
100 000-149 000	0.68 (0.48-0.97)	0.61 (0.48-0.79)	
150 000-199 999	0.68 (0.47-1.01)	0.62 (0.45-0.84)	
200 000-299 999	0.64 (0.43-0.95)	0.58 (0.43-0.79)	
300 000-499 999	0.38 (0.25-0.58)	0.34 (0.23-0.49)	0.36 (0.28-0.46)
500 000-999 999	0.43 (0.29-0.64)	0.39 (0.28-0.54)	
≥1 000 000	0.41 (0.24-0.69)	0.37 (0.24-0.58)	

Abbreviation: HR, hazard ratio.

^a^ The main effect for Black represents the Black-White racial differential in mortality at age 20 years. The HR for other ages can be computed as follows: HR_Black_  × (HR_Age × Black_)^(Age − 20 years)^, where HR_Black_ represents the main effect and HR_Age x Black_ represents the interaction term. For example, the HR for the Black-White racial differential based on model 3 would be 1.91 (4.76 × 0.97^30^) at age 50 years and 0.89 at age 75 years.

^b^ Refers to Asian or Pacific Islander; multiracial; Native American, Aleutian Islander, or Eskimo; and other.

^c^ Wealth is expressed in 1995 dollars.

Table 2 shows results from models fit separately for mortality before and after age 65 years. The top half of the table compares the demographic-adjusted mortality disparities for the bottom vs the top category of wealth with the corresponding disparities by other SES-related measures and smoking history. The HRs for all SES measures appeared to diminish with age, although the differences between the 2 age groups were significant only for household income.

**Table 2.** Demographic Adjusted and Fully Adjusted HRs for SES Disparities in Mortality Before and After Age 65 Years, Midlife in the United States Study, 1995-2013^a^

Model and variable	HR (95% CI)	
	Age 20-64 y	Age 65-92 y
Demographic adjusted^b^		
Childhood SES (≤30th percentile vs >90th percentile)	1.69 (1.04-2.76)	1.21 (0.84-1.74)
Education (high school graduate or less vs master’s degree or higher)	2.63 (1.61-4.30)	1.56 (1.17-2.07)
Occupational SEI (<29.8 vs ≥60)	2.02 (1.29-3.18)	1.50 (1.13-1.99)
Household income (<$35 000 vs ≥$165 000)	3.15 (1.95-5.11)	2.01 (1.32-3.06)
Wealth ($0 or debt vs ≥$300 000)	3.33 (1.76-6.30)	2.69 (2.00-3.62)
Overall SES (≤30th percentile vs >90th percentile)	3.55 (2.06-6.13)	2.15 (1.55-2.97)
Smoking history (current vs never smoker)	3.04 (2.30-4.00)	3.50 (2.84-4.32)
Fully adjusted^c^		
Education (high school graduate or less vs master’s degree or higher)	1.43 (0.84-2.43)	1.22 (0.88-1.67)
Occupational SEI (<29.8 vs ≥60)^d^	1.01 (0.60-1.71)	1.14 (0.81-1.60)
Household income (<$35 000 vs ≥$165 000)^e^	1.45 (0.83-2.53)	1.17 (0.76-1.81)
Wealth ($0 or debt vs ≥$300 000)^e^	1.66 (0.83-3.30)	1.89 (1.33-2.67)
Overall SES (≤30th percentile vs >90th percentile)	1.32 (0.72-2.42)	1.46 (1.02-2.09)
Smoking history (current vs never smoker)^f^	2.02 (1.49-2.75)	3.05 (2.41-3.85)
No. of observations^g^	5589	2884

Abbreviations: HR, hazard ratio; SEI, socioeconomic index; SES, socioeconomic status.

^a^ Here, we used the 4-category version of wealth (Table 1, model 3), and for comparability, the other SES measures were categorized to have a similar distribution, or as close as possible given the level of measurement (see eTable 1 in the Supplement).

^b^ Each row represents a separate model that includes the specified variable controlling only for age (as the time metric), sex, and race. To test whether the HR differs between the 2 age intervals, we refit the model for each SES measure to the pooled data across all ages and include interactions between each factor and the age intervals (ie, 20-64 vs 65-92 years). Those interactions were jointly significant only for household income (data not shown); that is, income is the only SES measure for which there is strong evidence that the disparity decreases with age.

^c^ Each row represents a separate model that includes the specified variable controlling for all potential confounders listed in eTable 2 in the Supplement, including childhood SES as a continuous variable.

^d^ This model also includes education as a potential confounder.

^e^ This model also includes education and occupational SEI as potential confounders.

^f^ This model also includes the composite measure of overall SES as a potential confounder.

^g^ A given respondent may contribute exposure to both age intervals (eg, a women aged 60 years at baseline in 1995 who survived to the end of follow-up in 2013, when she would have reached age 78 years, would contribute exposure at ages 60-64 years for the model of mortality before 65 years and exposure at ages 65-78 years for mortality after age 65 years).

Before age 65 years, the HR for wealth was larger than the HR for any other single SES-related measure (Table 2). The disparity by household income was also large, but disparities by education and occupational SEI were smaller. Not surprisingly, the HR for childhood SES was smaller than the HRs for more proximate measures of current SES. The demographic-adjusted disparity was largest for the composite measure of overall SES (HR, 3.55; 95% CI, 2.06-6.13).

eFigure 1 in the Supplement shows the estimated probability of surviving from age 25 to 65 years by SES measures and smoking history. There was a 12 percentage point differential in survival between the bottom (82%) vs the top category of household income (94%). The survival differentials between the lowest and highest categories were somewhat smaller for wealth (86% vs 95%), education (86% vs 95%), and occupation (86% vs 93%), and the survival differential was smallest for childhood SES (87% vs 92%).

After age 65 years, the demographic-adjusted wealth disparity was much larger (HR, 2.69; 95% CI, 2.00-3.62) than the disparities by any other SES-related measure (Table 2). As shown in eFigure 2 in the Supplement, there was a 31 percentage point difference in the probability of surviving from age 65 to 85 years between those with no assets or debt (40%) vs those with at least $300 000 (71%). The gaps by other SES-related measures were much smaller (13-21 percentage points).

Wealth matters, but the association of smoking with mortality was larger than those for any of the SES measures: the demographic-adjusted mortality rate among current smokers was more than 3 times that of never smokers (Table 2). Before age 65 years, the differentials in survival by smoking history were only slightly larger than those for income disparities (eFigure 1 in the Supplement), but at older ages, the smoking differential exceeded the SES disparities (eFigure 2 in the Supplement). There was a 42 percentage point difference in the probability of surviving from age 65 to 85 years between current (24%) and never smokers (66%), whereas the differential between the poorest and wealthiest categories was 31 percentage points (40% vs 71%).

In the fully adjusted models (Table 2), the SES-associated disparities were much smaller, suggesting that a substantial share of the differentials resulted from selection and/or reverse causality, and most were no longer significant. For example, part of the mortality differential may be because severe illness decimates wealth. Hospitalizations within the past 12 months were associated with higher mortality, and a costly hospitalization can adversely affect the respondent’s current level of wealth. Among respondents with 3 or more hospitalizations, 52% (31 respondents) reported zero assets vs only 29% (1625 respondents) of those with no recent hospitalization. Once we controlled for hospitalizations, the association between wealth and mortality became notably smaller (ie, perhaps because of endogeneity). The wealth differential in survival may also result from the selection process that determines wealth (ie, wealth is not randomly distributed). For example, married individuals tend to have more wealth^25^ and exhibit lower mortality^26^ than those who are not married. At baseline, 14% (606 respondents) of the married respondents in the MIDUS had at least $300 000 in assets, whereas the corresponding percentage was only 5% (32 respondents) for those who had never married. Part of that differential results from the definition of the measure, which includes assets of the spouse. Consequently, adjustment for marital status decreases the association between wealth and mortality. We found a similar pattern for smoking history: current smokers had less wealth but higher mortality than their nonsmoking counterparts.

After adjusting for all observed confounders, there was still a notable wealth disparity in mortality after age 65 years (HR, 1.89; 95% CI, 1.33-2.67), but the smoking disparity was even larger. After age 65 years, mortality rates were more than 3 times higher for current smokers than for never smokers; before age 65 years, the HR was smaller (2.02; 95% CI, 1.49-2.75).

**Figure 2.**
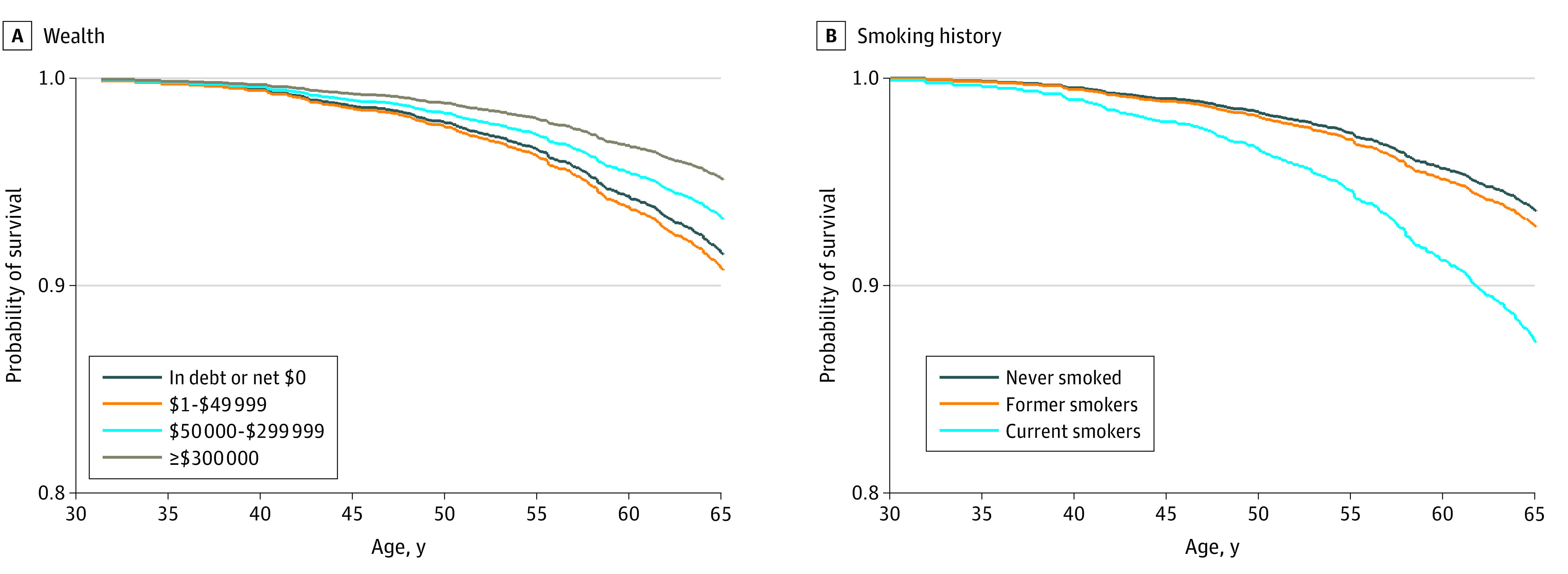
Fully Adjusted Probability of Surviving From Age 25 to 65 Years by Wealth and Smoking There were no deaths before age 30 years. The estimated survival curves are based on a model of age-specific mortality before age 65 years regressed on (A) wealth (in 4 categories) and (B) smoking history, controlling for all other potential confounders (ie, all covariates listed in eTable 2 in the Supplement; the model for wealth also includes education and occupational socioeconomic index, while the model for smoking includes the composite measure of overall socioeconomic status). All covariates except the specified variable (ie, wealth or smoking) are fixed at the mean for the sample.

**Figure 3.**
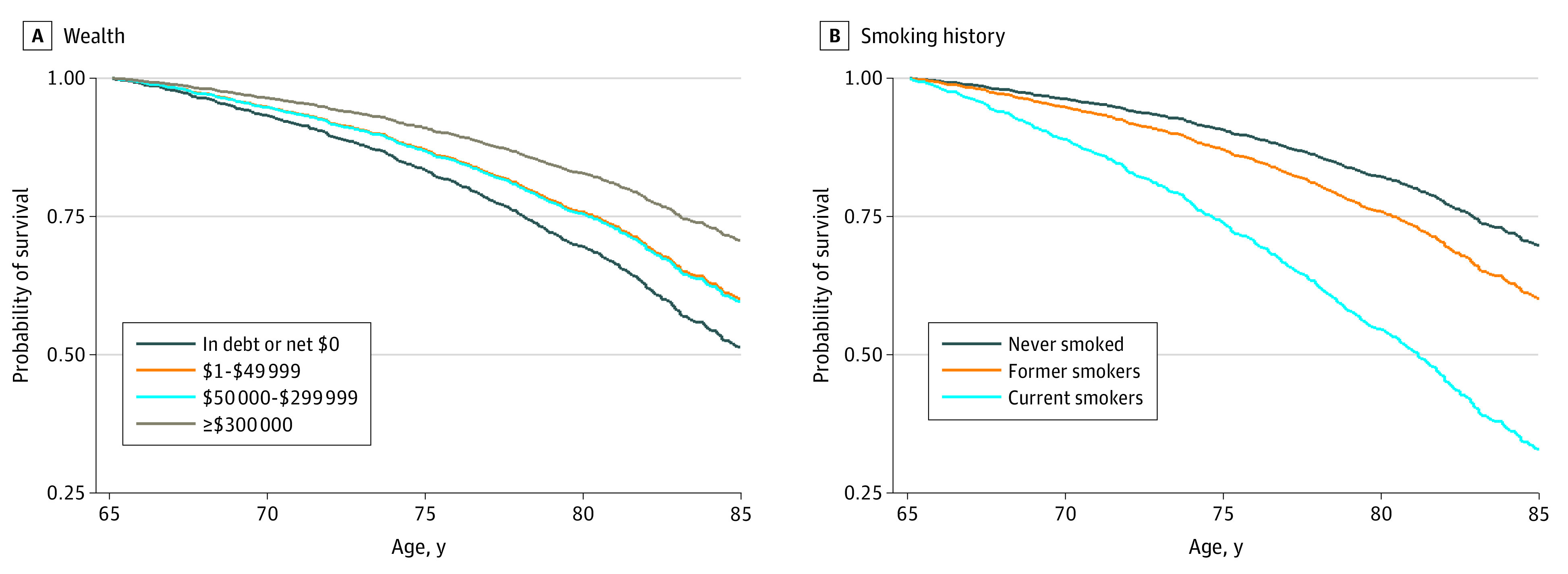
Fully Adjusted Probability of Surviving From Age 65 to 85 Years by Wealth and Smoking The estimated survival curves are based on a model of age-specific mortality after age 65 years regressed on (A) wealth (in 4 categories) and (B) smoking history, controlling for all other potential confounders (ie, all covariates listed in eTable 2 in the Supplement; the model for wealth also includes education and occupational socioeconomic index, while the model for smoking includes the composite measure of overall socioeconomic status). All covariates except the specified variable (ie, wealth or smoking) are fixed at the mean for the sample.

Figure 2 and Figure 3 show the absolute differentials in the survival probabilities (from age 25 to 65 years and 65 to 85 years) by wealth vs smoking history based on the fully adjusted model. The smoking differentials were far greater than the wealth differentials. For example, the probability of surviving from age 65 to 85 years was 19 percentage points higher among those with at least $300 000 in wealth (70%) than for those with debt or no assets (51%); in contrast, the differential between never smokers (70%) and current smokers (33%) was 37 percentage points (Figure 3).

## Discussion

There are many previous studies of wealth and mortality based on the Health and Retirement Survey,^4,7,8,11,27,28^ but they are limited to US individuals older than 50 years, who may have already bought a home and begun accumulating wealth. We know of 2 other studies based on the Panel Study of Income Dynamics: the first^13^ was restricted to US individuals aged 45 years and older, and the second^6^ did not evaluate whether the association between wealth and mortality varies by age.

In this cohort study, MIDUS offers the opportunity to investigate the association between wealth and mortality among younger US individuals (ie, 36% of the sample was aged 40 years or younger at baseline). In this life stage, individuals are developing their career and starting to accumulate wealth. Although mortality rates remain low at these young ages, a report by the National Academy of Sciences, Engineering, and Medicine^29^ concluded that increased mortality among working-age US individuals was the main reason for the 3-year consecutive decline in US life expectancy during 2015 to 2017. As they note, “Declining economic conditions tend to weaken societal institutions, community resources, family bonds, social networks, and access to health care—all of which could help explain disparities in working-age mortality according to race and ethnicity, socioeconomic status, and geography.”^30^

The demographic-adjusted estimates quantify the magnitude of the mortality disparity associated with wealth, but that does not necessarily mean that wealth has a causal effect on longevity. A recent study concluded that, “policies to support individuals’ ability to accrue wealth and to achieve financial security in adulthood could have considerable health benefits.”^5^ That would be true only if wealth has a causal effect and is not merely a result of selection and the detrimental effect of illness on wealth. Nonetheless, even if the effect is not causal, Smith points out that, “Whatever the origins of this stratification, it has profound implications for population health, where the consequences are serious and where the core reasons remain a mystery.”^31^

### Limitations

This analysis has 4 main limitations. First, although we controlled for a wider range of confounders than previous studies, we could not rule out potential endogeneity. Second, baseline measures of both wealth and health status did not allow us to determine clear temporal ordering (eg, did ill health decimate wealth or did financial insecurity contribute to chronic illness?). Third, there is potential bias resulting from nonresponse and misreporting. Younger individuals, men, those from racial minority groups, those who were less educated, and unmarried persons were less likely to complete the self-administered questionnaire. With the exception of men, those subgroups tend to have less wealth. Income is also notoriously susceptible to misreporting. Richer individuals are more likely than poor people to underreport their income on surveys.^32^ If a similar pattern holds true for reporting wealth, it could bias our results. Fourth, given the small percentage of individuals from racial minority groups in the sample, we have little statistical power to investigate racial differences in the association between wealth and mortality, and the first wave of MIDUS did not include self-reported ethnicity.

## Conclusions

In this cohort study, we found no longevity differential associated with additional wealth beyond $500 000 (in 1995 dollars). Even after adjusting for potential confounders, there is still a sizeable wealth disparity in mortality after age 65 years, although the smoking differential is far larger and is evident across all ages 25 and older. Health care practitioners cannot modify their patient’s wealth, but they should continue to discourage smoking. Wealth may be associated with longevity, but just don’t smoke.

## Notes

Supplement. **eAppendix 1.** Mortality Follow-up **eAppendix 2.** SES-Related Measures **eAppendix 3.** Multiple Imputation Procedures **eTable 1.** Descriptive Statistics for SES-Related Measures, MIDUS 1995-96 (N=6,320) **eTable 2.** Descriptive Statistics for Potential Confounders, MIDUS 1995-96 (N=6,320) **eFigure 1.** Probability of Surviving From Age 25 to 65 by SES Measures and Smoking History, Demographic-Adjusted **eFigure 2.** Probability of Surviving From Age 65 to 85 by SES Measures and Smoking History, Demographic-Adjusted
eReferences
