# Supplementary material for: Assessment of Mortality Disparities by Wealth Relative to Other Measures of Socioeconomic Status Among US Adults
Source: JAMA Netw Open. 2022 Apr 8;5(4):e226547. doi: 10.1001/jamanetworkopen.2022.6547 (PMC8994125; doi:10.1001/jamanetworkopen.2022.6547)
Supplement: Supplement. — eAppendix 1. Mortality Follow-up eAppendix 2. SES-Related Measures eAppendix 3. Multiple Imputation Procedures eTable 1. Descriptive Statistics for SES-Related Measures, MIDUS 1995-96 (N=6,320) eTable 2. Descriptive Statistics for Potential Confounders, MIDUS 1995-96 (N=6,320) eFigure 1. Probability of Surviving From Age 25 to 65 by SES Measures and Smoking History, Demographic-Adjusted eFigure 2. Probability of Surviving From Age 65 to 85 by SES Measures and Smoking History, Demographic-Adjusted eReferences [file jamanetwopen-e226547-s001.pdf]

Supplemental Online Content

Glei DA, Lee C, Weinstein M. Assessment of mortality disparities by wealth relative to other measures of socioeconomic status among US adults. *JAMA Netw Open*. 2022;5(4):e226547. doi:10.1001/jamanetworkopen.2022.6547

eAppendix 1. Mortality Follow-up
eAppendix 2. SES-Related Measures
eAppendix 3. Multiple Imputation Procedures
eTable 1. Descriptive Statistics for SES-Related Measures, MIDUS 1995-96 (N=6,320)
eTable 2. Descriptive Statistics for Potential Confounders, MIDUS 1995-96 (N=6,320)
eFigure 1. Probability of Surviving From Age 25 to 65 by SES Measures and Smoking History, Demographic-Adjusted
eFigure 2. Probability of Surviving From Age 65 to 85 by SES Measures and Smoking History, Demographic-Adjusted
eReferences

This supplemental material has been provided by the authors to give readers additional information about their work.

© 2022 Glei DA et al. *JAMA Network Open.*

eAppendix 1. Mortality Follow-up

Vital status was based on: 1) searches of the National Death Index (NDI); 2) Wave 3 tracing (conducting between May 2013 and 2017), and 3) longitudinal sample maintenance.[1] The most recent mortality file for MIDUS includes deaths that occurred as late as March 2018. However, the Wave 3 fieldwork began in May 2013. Thus, we suspect mortality follow-up is incomplete beyond May 31, 2013 (e.g., there is no way to know whether someone who was interviewed in May 2013 was still alive at some later date unless they rechecked vital status). The last NDI search that included all MIDUS survivors covered the period through December 31, 2009. MIDUS conducted a more recent search of the NDI through the end of 2016, but they included only 713 respondents (out of 5,806 MIDUS respondents thought to be still alive). For this search, they selected MIDUS respondents for whom they had sufficient identifiers for matching and who they suspected might be deceased (e.g., no recent contact, invalid addresses, etc.).[*]

An examination of the mortality rates by sex, age, and calendar year provides further evidence suggesting that mortality follow-up is incomplete beyond 2013. We estimated age-specific mortality using a Cox model with age the time metric and controlling for sex and calendar year dummies (as a time-varying covariate). Compared with 1995, the age-specific mortality rates did not differ significantly for any year between 1996 and 2014. However, mortality rates began to decline precipitously in 2015 (HR=0.70*, p<.05, relative to 1995) and continued to decline in every subsequent year (HR=0.47, p<0.001, in 2016; HR=0.25, p<0.001, in 2017; and HR=0.017, p<0.001, in 2018). Mortality decline of this magnitude is particularly suspicious given that, among the US national population, life expectancy actually *decreased* (i.e., overall mortality increased) for three consecutive years between 2014 and 2017. There is no reason to expect the mortality rates among the MIDUS cohort to decline so dramatically after 2014. We believe that most (if not all) of the apparent mortality decline is a statistical artifact resulting from incomplete mortality follow-up. Thus, we have restricted our analysis to mortality through May 2013.

Finegood et al.[2] modeled mortality through October, 2018,[†] but we suspect that their estimates are attenuated because of incomplete mortality follow-up during the last five years of that period. As a sensitivity check, we refit Model 1 from Table 1 for mortality through 12/31/2016. The HRs for wealth (e.g., HR=0.51, 95% CI 0.35-0.74 for those with $1M+ in wealth relative to those in debt) are weaker (i.e., closer to 1.0) than those shown in Table 1 (e.g., HR=0.41, 95% CI 0.24-0.69 for $1M+ vs. those in debt), but they follow a similar pattern.

---

[*] It is unclear how "recent contact" was defined. The documentation states that they excluded respondents who had been recently contacted via participation in MIDUS projects or who were identified as alive during Wave 3 refielding in 2017.[1] However, the refielding included only those respondents who did not complete the self-administered questionnaire (SAQ) or cognitive component in 2015. Among the original MIDUS cohort, 3294 respondents were re-interviewed for Wave 3 between May 2013 and June 2014 (only 1 respondent was interviewed in May, 1527 were interviewed in June-July 2013; 94% were interviewed by 12/31/2013). We do not know whether they rechecked vital status at a later date for those individuals or whether they were presumed to be still alive. Information regarding the date when the SAQ was completed is not provided on the public-use file.

[†] It remains unclear why they presumed mortality follow-up continued through October 2018 even though the latest death occurred in March 2018; there were only 2 deaths in 2018 (one in February and one in March). In contrast, there were 31 deaths recorded in 2017 among the MIDUS cohort, 70 in 2016, and 76-77 deaths each year between 2012 and 2014.

© 2022 Glei DA et al. *JAMA Network Open.*

eAppendix 2. SES-Related Measures

Education

Educational attainment was measured in terms of degree completion ("What is the highest grade of school or year of college you completed?") using the following 12 response categories:

1=No school/some grade school (1-6);
2=Eighth grade/junior high school (7-8);
3=Some high school (9-12 no diploma/no GED);
4=GED;
5=Graduated from high school;
6=1 to 2 years of college, no degree yet;
7=3 or more years of college, no degree yet;
8=Graduated from a two-year college or vocational school, or associate's degree;
9=Graduated from a four- or five-year college, or bachelor's degree;
10=Some graduate school;
11=Master's degree; and
12=Ph.D., Ed.D., MD, DDS, LLB, LLD, JD, or other professional degree.

Occupational Socioeconomic Index (SEI)

The occupational SEI was created by Hauser and Warren[3] based on the three-digit 1980 census occupational codes. Scores range from 7.1 (shoe machine operator) to 80.5 (physician).

Household income

Income from each source (i.e., wages/salary, social security, government assistance, and all other sources such as pensions, investments, child support, or alimony) was reported in categories, which we recoded to the mid-point of the range within each category. Income from each source was top-coded at $200,000 (except government assistance, which was top-coded at $50,000). We recoded top-coded values to the harmonic mean of a Pareto distribution. As suggested by von Hippel et al.,[4] we compute the harmonic mean of a Pareto distribution with α equal to the maximum of one or $[\ln(n_{B-1} + n_B) - \ln(n_B)] \div [\ln(l_B) - \ln(l_{B-1})]$, where n_B is the number of cases in the top category; n_{B-1} is the number of cases in the penultimate category; l_B is the lower bound of the top category; and l_{B-1} is the lower bound of the penultimate category. Restricting α to a minimum of one ensures that the value of the top category is no greater than twice the lower bound of that category. We then summed across all sources to compute total income. We were unable to make an equivalence adjustment based on household size and composition because MIDUS did not collect that information at Wave 1.

© 2022 Glei DA et al. *JAMA Network Open.*

Assets

The self-administered questionnaire included the following questions, which we used to determine the current net wealth of the respondent and his/her spouse (if applicable).

J14. Suppose you (and your spouse or partner) cashed in all your checking and savings accounts, stocks and bonds, real estate, sold your home, your vehicles, and all your valuable possessions. Then suppose you put that money toward paying off your mortgage and all your other loans, debts, and credit cards. Would you have any money left over after paying your debts or would you still owe money?

1. **Would have money left over**
2. **Would still owe money**
3. **Debts would just about equal assets**

J15. How much would that be (that you had left over, or would owe)? Again, please write down the correct letter from the list on the previous page. (Your best estimate is fine. If your debts would just about equal your assets, enter "B".)

________________ **MONEY LEFT OVER/MONEY OWED (LETTER FROM LIST)**

List of Categories shown on previous page (above J8):

A.	Less than $0 (Loss)	M.	$10,000 - $10,999	AA.	$30,000 - $34,999
B.	$0 (None)	N.	$11,000 - $11,999	BB.	$35,000 - $39,999
C.	$1 - $1,000	P.	$12,000 - $12,999	CC.	$40,000 - $44,999
D.	$1,000 - $1,999	R.	$13,000 - $13,999	DD.	$45,000 - $49,999
E.	$2,000 - $2,999	S.	$14,000 - $14,999	EE.	$50,000 - $74,999
F.	$3,000 - $3,999	T.	$15,000 - $15,999	FF.	$75,000 - $99,999
G.	$4,000 - $4,999	U.	$16,000 - $16,999	GG.	$100,000 - $149,999
H.	$5,000 - $5,999	V.	$17,000 - $17,999	HH.	$150,000 - $199,999
I.	$6,000 - $6,999	W.	$18,000 - $18,999	II.	$200,000 - $299,999
J.	$7,000 - $7,999	X.	$19,000 - $19,999	JJ.	$300,000 - $499,999
K.	$8,000 - $8,999	Y.	$20,000 - $24,999	KK.	$500,000 - $999,999
L.	$9,000 - $9,999	Z.	$25,000 - $29,999	LL.	$1,000,000 or more

For J15, we recoded the responses to the mid-point of the range within each category, with those who reported themselves to be in debt on J14 coded as a negative number. The top category ($1,000,000+) was recoded to the harmonic mean of a Pareto distribution as described above for income.

Inflation adjustment for income and assets

For interviews conducted in 1996, we converted income and assets to 1995 dollars using the Consumer Price Index (CPI) provided by the Bureau of Labor Statistics (https://data.bls.gov/cgi-bin/cpicalc.pl). The median month among interviews completed in 1995 was April (which is treated as the reference), and the median month for those conducted in 1996 was July. Thus, the multiplier was 0.97 for data collected in 1996.

© 2022 Glei DA et al. *JAMA Network Open.*

Adult SES

The composite measure of overall SES was based on six variables measured at baseline: educational attainment and occupational SEI of the respondent (and, if applicable, his/her spouse or partner), annual household income, and current net assets of the respondent and spouse/partner combined. A similar measure has been used in prior studies.[5–9]

Because income and assets were positively skewed, we applied a square root transform to those two items. [If assets were negative, we used the untransformed values (i.e., everyone with net $0 assets or debt was assigned to zero on the transformed variable).] We standardized the six items and computed the average across relevant items (e.g., six items if married/partnered and both respondent and spouse/partner have ever been employed; three items if not married/partnered and respondent has never been employed; Cronbach's α=0.77 at baseline).

Childhood SES

The index for childhood SES was based on baseline measures of Mother's and Father's educational attainment and occupational SEI[3] as well as perceived financial status (i.e., respondent's rating their family's financial status relative to others, coded on a 7-point scale ranging from "a lot worse off" to "a lot better off"). We computed the average across the five standardized items (Cronbach's α=0.78).

© 2022 Glei DA et al. *JAMA Network Open.*

eAppendix 3. Multiple Imputation Procedures

Among the 6,320 respondents included in the analysis, the predictors with the highest percentage of missing data were assets (11%), household income (10%), waist circumference (7%), and father's health status when the respondent was age 16 (5%). We used the "ice" command to perform multiple imputation. For the multiple imputation process, we used information for all the analysis variables as well as whether the respondent ever drank alcohol regularly and wage/salary income for the respondent (and spouse/partner, if applicable).

For continuous variables, departures from normality may result in implausible imputations when using the default draw method. Assets, household income, and number of hospitalizations had a skewed distribution. Prior to imputation, we applied a square root transformation to the income variables, but that was not possible for assets because it has negative values. To ensure that imputed values were within the range of observed values, we used prediction matching for all those variables and several others for which imputation generated out of range values (i.e., age, education, occupational SEI score, and physical limitations).

For imputation of ordinal variables (e.g., self-assessed health status), we used an ordered logit model for imputation. We performed five imputations and then used the "mim" prefix command to re-estimate the model for each imputation and combine the five sets of estimates using Rubin's rules.[10]

© 2022 Glei DA et al. *JAMA Network Open.*

eTable 1. Descriptive statistics for SES-related measures,[a] MIDUS 1995-96 (N=6,320)

	No. (%)
Childhood SES	
≤ 30th percentile	1897 (30.0)
31st-60th percentiles	1895 (30.0)
61st-90th percentiles	1896 (30.0)
> 90th percentile	632 (10.0)
Education	
H.S. graduate or less	2390 (37.8)
Some college	1916 (30.3)
Bachelor's degree	1324 (20.9)
Master's degree or higher	691 (10.9)
Occupational SEI score	
Low (7.1-29.7)	1863 (29.5)
Medium (29.8-43.9)	1952 (30.9)
High (44-59.9)	1788 (28.3)
Very high (60-80.5)	717 (11.3)
Household Income (in 1995 dollars)	
Less than $35,000	1851 (29.3)
$35,000-64,999	1814 (28.7)
$65,000-164,999	2017 (31.9)
$165,000 or more	638 (10.1)
Wealth (in 1995 dollars)	
In debt	834 (13.2)
Net $0	1034 (16.4)
$1-49,999	1856 (29.4)
$50,000-99,999	710 (11.2)
$100,000-149,000	516 (8.2)
$150,000-199,999	320 (5.1)
$200,000-299,999	328 (5.2)
$300,000-499,999	326 (5.2)
$500,000-999,999	258 (4.1)
$1,000,000 or more	137 (2.2)
Overall SES	
≤ 30th percentile	1896 (30.0)
31st-60th percentiles	1896 (30.0)
61st-90th percentiles	1896 (30.0)
> 90th percentile	632 (10.0)

[a] See eAppendix 2 for more information about the SES-related measures. We initially categorized wealth into the 10 categories shown above, which retains the first two response categories (i.e., in debt, net $0) and all the response categories above $100,000 from the original question (see p. 4). However, we collapsed the remaining 28 categories into two groups ($1-49,999 and $50,000-99,999). For the models presented in Table 2, we used the simplified 4-category version of wealth (i.e., In debt/Net $0; $1-49,999; $50,000-299,999; $300,000+), and for comparability, the other SES measures were categorized to have a similar distribution—or as close as possible given the level of measurement.

© 2022 Glei DA et al. *JAMA Network Open.*

eTable 2. Descriptive Statistics for Potential Confounders, MIDUS 1995-96 (N=6,320)

	Statistics
Demographic	
Age, mean (SD)	46.9 (12.9)
Female, No. (%)	3318 (52.5)
Black/African American, No. (%)	342 (5.4)
White, No. (%)	5709 (90.3)
Other race, No. (%)[a]	269 (4.3)
Socioeconomic	
Childhood SES index (-2.5 to 3.5), mean (SD)[b]	0.0 (1.0)
Married, No. (%)	4269 (67.6)
Partnered, No. (%)	299 (4.7)
Widowed, No. (%)	305 (4.8)
Divorced/separated, No. (%)	832 (13.2)
Never married, No. (%)	615 (9.7)
Covered by health insurance, No. (%)	5661 (89.6)
Currently employed, No. (%)	4645 (73.5)
Retired, No. (%)	809 (12.8)
Neither employed nor retired, No. (%)	866 (13.7)
Health-Related	
Mom deceased when respondent was age 16, No. (%)	246 (3.9)
If alive: Mom's health when respondent was age 16 (1-5, 5=Excellent), median (IQR)	4 (1)
Dad deceased when respondent was age 16, No. (%)	420 (6.6)
If alive: Dad's health when respondent was age 16 (1-5, 5=Excellent), median (IQR)	4 (1)
History of heart trouble, No. (%)	822 (13.0)
History of cancer, No. (%)	458 (7.2)
Stroke in the past 12 months, No. (%)	45 (0.7)
Diabetes in the past 12 months, No. (%)	325 (5.1)
Respiratory/lung problems in past 12 months, No. (%)	899 (14.2)
Hypertension in the past 12 months, No. (%)	1115 (17.6)
Arthritis, rheumatism, or other bone/joint disease in the past 12 months, No. (%)	1241 (19.6)
Self-assessed health status (1-5, where 5=excellent), median (IQR)	4 (1)
Number of hospitalization in the past 12 months (0-10), median (IQR)	0 (0)[c]
Number of physical limitations (0-8), median (IQR)[d]	1 (4)
Body mass index (BMI) < 18.5, No. (%)	117 (1.9)
BMI 18.5-24.9, No. (%)	2497 (39.5)
BMI 25-29.9, No. (%)	2370 (37.5)
BMI 30-34.9, No. (%)	891 (14.1)
BMI 35+, No. (%)	445 (7.0)
Waist circumference (35.6-167.6 cm), mean (SD)	89.9 (14.7)
If married/partnered: Spouse's health status (1-5, 5=Excellent), median (IQR)	4 (1)
Substance use	
Never smoked, No. (%)	3078 (48.7)
Former smoker, No. (%)	1867 (29.5)
Current smoker, No. (%)	1375 (21.8)
Any alcohol abuse, No. (%)[e]	430 (6.8)
Any drug abuse, No. (%)[f]	429 (6.8)

IQR = Interquartile range

[a] Includes respondents who identified as Asian or Pacific Islander; multiracial; Native American or Aleutian Islander/Eskimo; and other.

[b] See eAppendix 2 for more information about the construction of this variable.

[c] The vast majority (89%) of the sample had no hospitalizations in the past 12 months.

[d] Physical limitations counts the number of the following physical tasks for which the respondent reports at least "a little" health limitation: 1) Lifting or carrying groceries; 2) climbing several flights of stairs; 3) bending, kneeling, or stooping; 4) walking more than a mile; 5) walking several blocks; 6) walking one

© 2022 Glei DA et al. *JAMA Network Open.*

block; 7) vigorous activity (e.g., running, lifting heavy objects); and 8) moderate activity (e.g., bowling, vacuuming).

[e] Alcohol abuse is based on four items from the Michigan Alcohol Screening Test (MAST),[11] which has been used in many prior studies.[5,7,12]

[f] Drug abuse is based on the Drug Dependence scale of the Composite International Diagnostic Interview Short Form (CIDI-SF)[13] and has also been used in prior studies.[5,7] MIDUS asked about the same types of drugs as the CIDI-SF (i.e., sedatives, tranquilizers, amphetamines, prescription painkillers, inhalants, marijuana/hashish, cocaine/crack/free base, hallucinogens, heroin, prescription anti-depressants), but the MIDUS question referred only to non-medical use (i.e., "on your own"—that is, "without a doctor's prescription, in larger amounts that prescribed, or for a long period than prescribed") whereas the CIDI-SF screener includes any use of those same drugs.

© 2022 Glei DA et al. *JAMA Network Open.*

eFigure 1. Probability of Surviving From Age 25 to 65 by SES Measures and Smoking History, Demographic-Adjusted

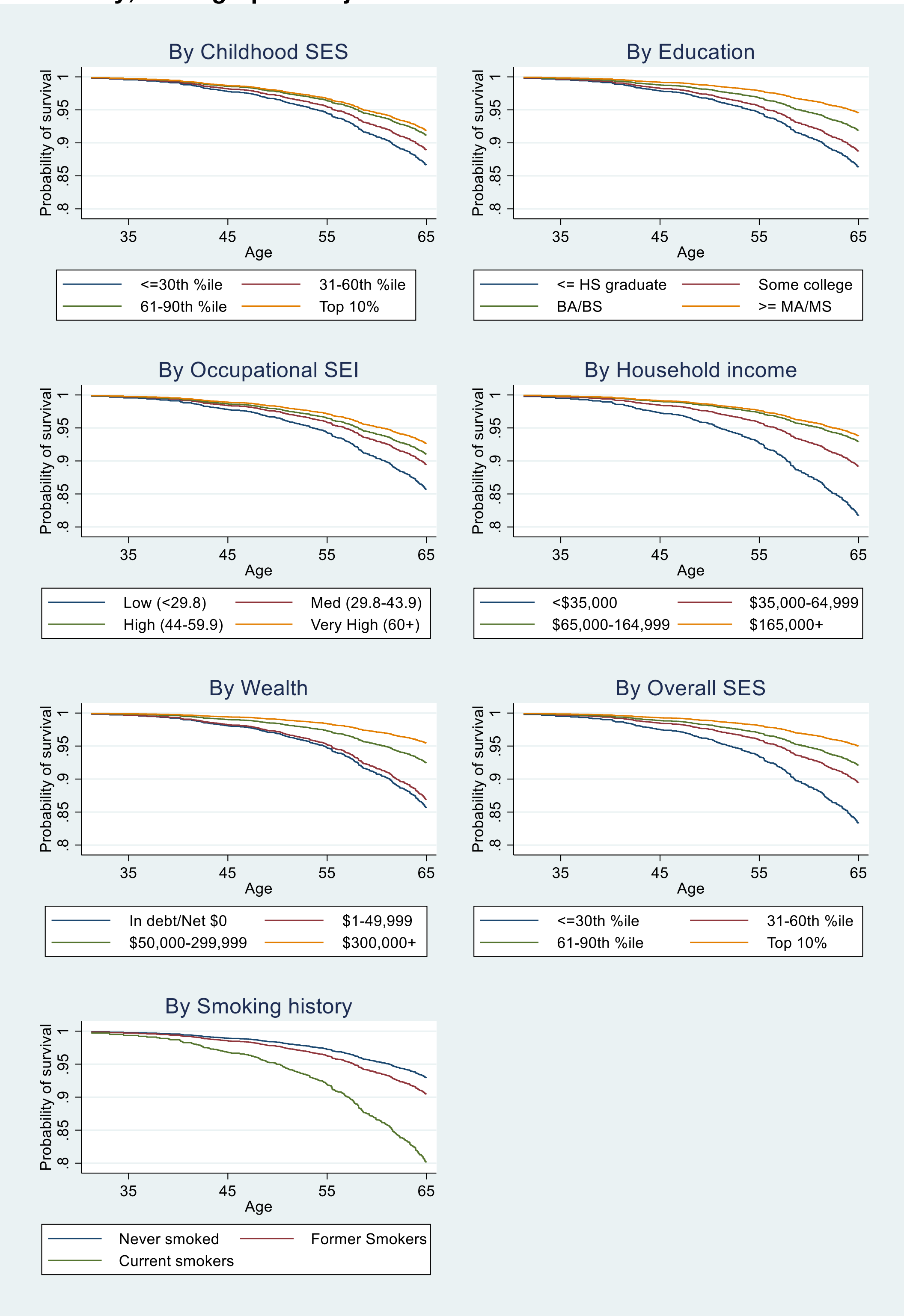

Note: There were no deaths below age 30. The predicted survival curves are based on a model of age-specific mortality below age 65 that controls for sex and race; those variables are fixed at the mean for the sample.

© 2022 Glei DA et al. *JAMA Network Open.*

eFigure 2. Probability of Surviving From Age 65 to 85 by SES Measures and Smoking History, Demographic-Adjusted

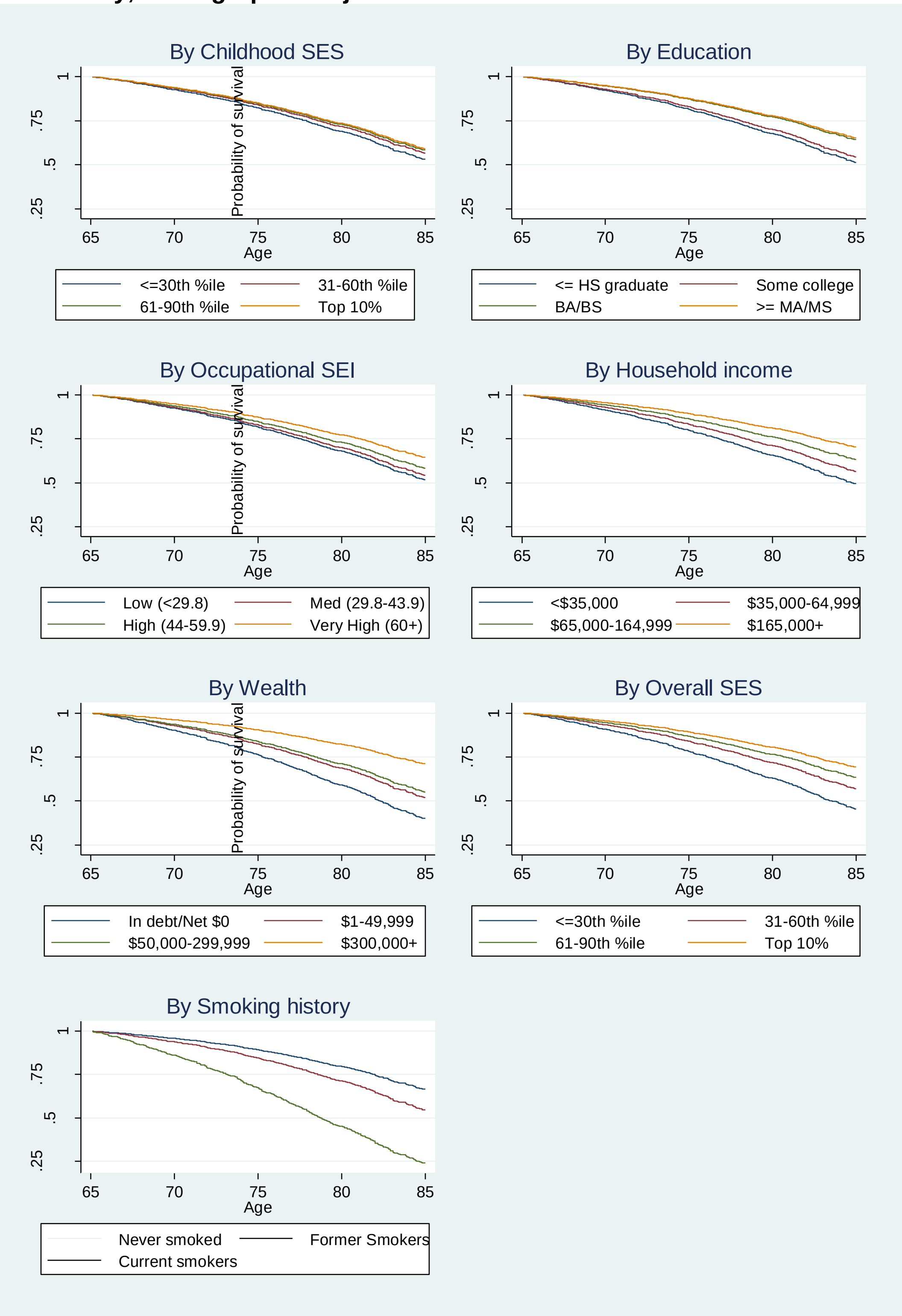

Note: The predicted survival curves are based on a model of age-specific mortality above age 65 that controls for sex and race; those variables are fixed at the mean for the sample.

© 2022 Glei DA et al. *JAMA Network Open.*

eReferences

1. Ryff C, Almeida D, Ayanian J, et al. Midlife in the United States: Core Sample Mortality Data, 2016: Version 3 (ICPSR 37237). Published online 2020. doi:10.3886/ICPSR37237.V3

2. Finegood ED, Briley DA, Turiano NA, et al. Association of Wealth With Longevity in US Adults at Midlife. *JAMA Health Forum*. 2021;2(7):e211652-e211652. doi:10.1001/jamahealthforum.2021.1652

3. Hauser RM, Warren JR. Socioeconomic Indexes for Occupations: A Review, Update, and Critique. *Sociological Methodology*. 1996;27(1):177-298. doi:https://doi.org/10.1111/1467-9531.271028

4. von Hippel PT, Scarpino SV, Holas I. Robust estimation of inequality from binned incomes. *Sociological Methodology*. 2016;46(1):212-251. doi:10.1177/0081175015599807

5. Glei DA, Weinstein M. Drug and alcohol abuse: the role of economic insecurity. *AmJHealth Behav*. 2019;43(4):838-853. doi:10.5993/AJHB.43.4.16

6. Glei DA, Goldman N, Weinstein M. A growing socioeconomic divide: Effects of the Great Recession on perceived economic distress in the United States. *PLoS One*. 2019;14(4):e0214947. doi:10.1371/journal.pone.0214947

7. Glei DA, Lee C, Weinstein M. Socioeconomic disparities in U.S. mortality: The role of smoking and alcohol/drug abuse. *SSM - Population Health*. 2020;12:100699. doi:10.1016/j.ssmph.2020.100699

8. Goldman N, Glei DA, Weinstein M. Declining mental health among disadvantaged Americans. *ProcNatlAcadSciUSA*. 2018;115(28):7290-7295. doi:10.1073/pnas.1722023115

9. Glei DA, Goldman N, Weinstein M. Perception has its own reality: subjective versus objective measures of economic distress. *Popul Dev Rev*. 2018;44(4):695-722. doi:10.1111/padr.12183

10. Royston P, Carlin JB, White IR. Multiple imputation of missing values: new features for mim. *Stata Journal*. 2009;9(2):252-264.

11. Selzer ML. The Michigan alcoholism screening test: the quest for a new diagnostic instrument. *AmJPsychiatry*. 1971;127(12):1653-1658. doi:10.1176/ajp.127.12.1653

12. Ransome Y, Slopen N, Karlsson O, Williams DR. Elevated inflammation in association with alcohol abuse among Blacks but not Whites: results from the MIDUS biomarker study. *JBehavMed*. 2018;41(3):374-384. doi:10.1007/s10865-017-9905-4

13. Kessler RC, Andrews G, Mroczek D, Ustun TB, Wittchen HU. The World Health Organization Composite International Diagnostic Interview Short Form (CIDI-SF). *Int J Methods Psychiatr Res*. 1998;7(4):171-185.

© 2022 Glei DA et al. *JAMA Network Open.*
